# Effects on the Facial Growth of Rapid Palatal Expansion in Growing Patients Affected by Juvenile Idiopathic Arthritis with Monolateral Involvement of the Temporomandibular Joints: A Case-Control Study on Posteroanterior and Lateral Cephalograms

**DOI:** 10.3390/jcm9041159

**Published:** 2020-04-18

**Authors:** Cinzia Maspero, Davide Cavagnetto, Andrea Abate, Paolo Cressoni, Marco Farronato

**Affiliations:** 1 Department of Biomedical, Surgical and Dental Sciences, School of Dentistry, University of Milan, 20100 Milan, Italy; davide.cavagnetto@gmail.com (D.C.); andreabate93@gmail.com (A.A.); paolo.cressoni@gmail.com (P.C.); marco.farronato@unimi.it (M.F.); 2 Fondazione IRCCS Cà Granda, Ospedale Maggiore Policlinico, 20100 Milan, Italy

**Keywords:** juvenile arthritis, temporomandibular joint, palatal expansion technique, facial asymmetry, orthodontic appliances, growth and development

## Abstract

Background: Juvenile idiopathic arthritis (JIA) affecting temporomandibular joints (TMJ) in growing patients results in maxillofacial deformities, especially if only one condyle has been affected by the rheumatic disease. Mandibular hypoplasia is the most common issue and it may be associated with maxillary hypoplasia. The aim of this retrospective case-control study is to evaluate the effects of rapid maxillary expansion (RME) in these patients. Methods: 25 growing patients affected by maxillary hypoplasia, currently in a quiescent phase of JIA for at least one year and monolateral involvement of the TMJs, were treated with RME. Data gathered from posteroanterior and lateral cephalograms before and after 1 year from RME were compared to those of 25 non-JIA controls. Results: Nasal cavity width, maxillary width and upper and lower intermolar width statistically increased. Maxillary and mandibular symmetry indexes presented a statistically significant increase, so did the skeletal class. No signs or symptoms of TMJ activity of JIA occurred according to Research Diagnostic Criteria for Temporomandibular Disorders (RDC/TMD) criteria. No difference was found when comparing JIA and non-JIA patients apart from the better improvement of several mandibular symmetry indexes in the affected TMJ side of JIA patients. This event is allegedly due to a worse baseline asymmetry in JIA patients that underwent a bigger relative improvement after treatment. Conclusions: Results suggest that solving maxillary hypoplasia and, therefore, premature contacts are likely to have allowed mandibular repositioning and condylar growth. RME is a safe and effective solution that can substantially improve maxillary and mandibular symmetry in growing patients affected by JIA with TMJ involvement.

## 1. Introduction

Juvenile idiopathic arthritis (JIA) include a heterogeneous group of conditions of uncertain aetiology defined as the chronic inflammation of articular connective tissue for a minimum of 6 weeks, before the of age 16 [1]. Incidence and prevalence in the population range between 2 and 20, and between 16 and 150 per 100,000 subjects respectively [2,3]. JIA is, therefore, considered as one of the most common rheumatic diseases affecting children both in Europe and in North America [2,3].

The temporomandibular joint (TMJ) is involved in 38–72% of cases, depending on the JIA subtype examined and on the diagnostic method used [4,5,6]. TMJs can be affected bilaterally or unilaterally. Patients may present pain, functional disorders (i.e., difficulties in chewing and speaking), condylar and articular damages, bite force impairment and tenderness of masticatory muscles thus provoking severe disabilities [7,8,9,10]. TMJ involvement may cause relevant sagittal and vertical growth alterations in both jaws such as anterior open bite, severe micrognathia, marked asymmetry and posterior cross-bite [11,12]. Orthodontic treatment in JIA patients is based on a multidisciplinary approach with pharmacological and non-pharmacological interventions [2,13]. Orthodontic and gnathological treatment is important for early detection of TMJ involvement and for sagittal and vertical mandibular growth correction [6,14].

Medical literature on orthodontic treatment in patients suffering from this rheumatic condition is scarce [15]. Recent literature reviews have affirmed the importance of orthodontic treatment in growing JIA patients. No guidelines were proposed about the treatment protocols to follow [6,14,15,16,17]. A systematic review by Bremen and Ruf (2011) reported orthodontic treatments in growing subjects affected by JIA [6]. Treatment with functional appliances, such as Andresen activators or distraction splints, is recommended as it is reported to favour mandibular growth and to reduce mandibular asymmetry and intermaxillary divergence in non-JIA patients. JIA patients’ response to orthodontic functional treatment is indeed reduced compared to controls due to their rheumatic condition, and also differs between patients [18]. However, there is no agreement about interceptive orthodontic treatment effectiveness and orthognathic surgical correction of residual malocclusion at the end of growth is always an option [15].

As far as the authors are aware, there have been no previous studies which have evaluated rapid maxillary expansion in JIA patients. Effects of interceptive orthodontic treatment on the maxilla of JIA patients are not reported in literature. In 2017, Menezes et al. described a case of a patient treated with a quad-helix appliance in the maxilla and a Nance lingual arch in the mandible. However, they did not evaluate the effect on the maxilla apart from cross-bite correction [19]. JIA is associated with maxillary hypoplasia and, in particular with posterior cross-bite in deciduous and mixed dentition [20]. Growing patients affected by JIA with TMJ involvement might present maxillary hypoplasia and, therefore, could benefit from maxillary expansion with a rapid maxillary expander (RME), as it has been proven to be the treatment of choice for this condition in non-JIA patients [21,22,23]. No studies have described the use of this appliance in patients affected by JIA yet. Drawbacks of atraumatic orthodontic appliances, like Andresen activators, often reported in these patients’ treatments [15] include the limitations of dento-alveolar expansion compared to the skeletal expansion obtained with a RME, i.e., a slow movement of teeth inside the alveolar bone compared to a fast distraction osteogenesis induced by the separation of the palatine processes of the maxillary bones. In addition, the prolonged period of time when the slow transversal movement of maxillary posterior lateral teeth occurs it may cause premature contacts that deflect the mandible during functional movements, for example chewing, which are harmful to TMJ health [24,25,26]. RME is indeed a fast option for solving premature contacts and cross-bite within 15 to 21 days [27]. RME has been proven not to cause any damage to TMJs [28,29,30]. Positive effects of RME include repositioning of the mandible forward and an increase in condylar space, thus improving skeletal second class [31] and reducing condylar functional stresses [30].

RME have already been demonstrated to cause an augmentation of SNB angle, which measures the antero-posterior positioning of the mandible in relation to the cranial base (partly due to condylar repositioning inside the glenoid fossa and partly to condylar growth) [32], to reduce nasal resistances, and to improve nasal respiration and facial symmetry [33,34,35]. These positive effects would be also be desirable in the treatment of JIA patients because their most common facial traits, whenever TMJ are affected by this rheumatic condition, are mandibular hypoplasia and facial asymmetry [11,12]. The aim of this retrospective case-control study is to evaluate sagittal and transversal effects on maxillofacial structures of RME in JIA patients with no disease activity at the TMJ level for at least one year and only one TMJ affected by their rheumatic disease and to compare results with a non-JIA control group.

## 2. Experimental Section

### 2.1. Study Design and Ethical Approval

A retrospective case-control study was performed analyzing postero-anterior and lateral cephalograms before and after maxillary expansion of patients affected by JIA and comparing them with non-JIA controls.

The study protocol was approved by the Ethical Committee of the Fondazione IRCCS Ca’Granda, Ospedale Maggiore, Milan-Italy (protocol n.573/15). All patients’ parents provided written informed consent allowing the anonymous use of the patients’ medical records for research purposes regarding all the procedures performed. This study was performed according to the Declaration of Helsinki for human studies.

### 2.2. Type of Participants and Inclusion Criteria

The records of 25 Caucasian patients with a diagnosis of JIA with TMJ involvement (11 male and 14 females, mean age 10.3 ± 1.6) were selected from the archives of the Department of Biomedical Surgical and Dental Sciences, University of Milan, Italy. Inclusion criteria for the selection of patients affected by JIA were: diagnosis of JIA with unilateral TMJ involvement; transverse maxillary hypoplasia (severe crowding with no cross-bite, posterior unilateral or bilateral cross-bite) treated with a rapid maxillary expansion protocol with a hyrax type expander; treatment beginning during a JIA quiescent phase that lasted for at least 12 months; TMJ involvement classified as Grade 3 according to Cahill classification (juxta-articular erosions without any sign of an active inflammatory process in MRI scans) [36]; presence of lateral and postero-anterior radiographs before and after the expansion (mean distance between radiographs: 6 ± 5 months in JIA group and 6 ± 9 months in the end control group); records reporting the evaluation of TMJ involvement according to Research Diagnostic Criteria for Temporomandibular Disorders (RDC-TMD) IIIA criteria [37] before RME, at of maxillary expansion, and after 1 month, 3 months and 6 months; no previous orthodontic treatment. Methotrexate was taken by patients during the active phase of the pathology and they started treatment at the Department of Orthodontics, 12 months after their last dose of medication. Patients were evaluated according to the International League of Associations for Rheumatology (ILAR) criteria (8 cases polyarticular JIA, 17 oligoarticular JIA). The mean age of JIA onset was 7.1 years of age [38,39].

Inclusion criteria for the control group in order to obtain a clinically meaningful comparison were: transverse maxillary hypoplasia (severe crowding with no cross-bite, posterior unilateral or bilateral cross-bite) treated with a rapid maxillary expansion protocol with a hyrax type expander; skeletal class II, no malformation of the craniofacial area; no previous orthodontic treatment; presence of lateral and postero-anterior radiographs before and after expansion. The records of 25 non-JIA Caucasian patients matched with sex and age (9 male and 16 females, mean age 9.7 ± 1.4) treated with RME with the same diagnosis served as controls.

Exclusion criteria in both groups were congenital anomalies, dental anomalies (form, number and dimension of teeth), and previous orthodontic treatment.

All patients from both groups were treated with Hyrax palatal expander to correct maxillary transverse hypoplasia, to treat posterior cross bite and to increase the space within the arch, between 2013 and 2016 in the Orthodontic Department of Fondazione IRCCS Ca‘Granda Ospedale Maggiore Policlinico of Milan (University of Milan).

A Hyrax-type rapid maxillary expander was bonded to the maxillary second primary molars using a glass-ionomer orthodontic luting cement (Multi-Cure; Unitek, Monrovia, CA, USA). Two activations of the hyrax screw were performed by the orthodontist, and two by the patient’s parents as training immediately after appliance luting (0.25 mm per activation). On the following day, activations were prescribed twice a day (0.50 mm) until the next follow-up appointment after 7 days when patients were re-evaluated, and the clinician would decide whether to end or continue the activations. In the latter case a maximum of 14 additional activations would have been prescribed and patients would have been re-evaluated each for 7 days until the clinician decided to end activations. The therapeutic goal was achieved when the patients presented a dental overcorrection, that is when the contact between the palatal cusps of maxillary first permanent molars and the lower first permanent molars occurred at the edge of the lingual side of buccal cusps [40]. The active treatment lasted between 15 and 21 days. After that, the screw was fixed with a stainless steel ligature wire passing through the activation hole. The appliance was kept in place for at least 6 months to allow mineralization of the midpalatal sutures that had been opened.

### 2.3. Cephalometric Analysis

Teleradiographs in lateral and postero-anterior projection of all patients’ skulls were obtained from the same machine Orthophos XG(Sirona Group, Bensheim, Germany) with fixed focus sensor distance (150 cm), at the Dental and Maxillofacial Department of the Fondazione IRCCS Ca ´ Granda, Ospedale Maggiore Policlinico, Milan, Italy. Digital cephalometric tracings were performed by an operator using Dolphin Imaging software (Dolphin Imaging and Management Solutions; Los Angeles, CA, USA), which computed all reported measurements. Three weeks later, the tracings were made by a different operator and retraced with the first one in order to evaluate intra- and inter-operator variability on 15 randomly selected lateral and postero-anterior radiographs. Intraclass correlation coefficients (ICC) were calculated to compare intra- and inter-operator variability. Correlation coefficients for the skeletal measurements were greater than 0.92. Linear measurement errors averaged 0.4 mm (standard deviation (SD) 0.4 mm) and angular measurements averaged 0.6° (SD 0.5°). Method error was considered negligible.

**Figure 1:**
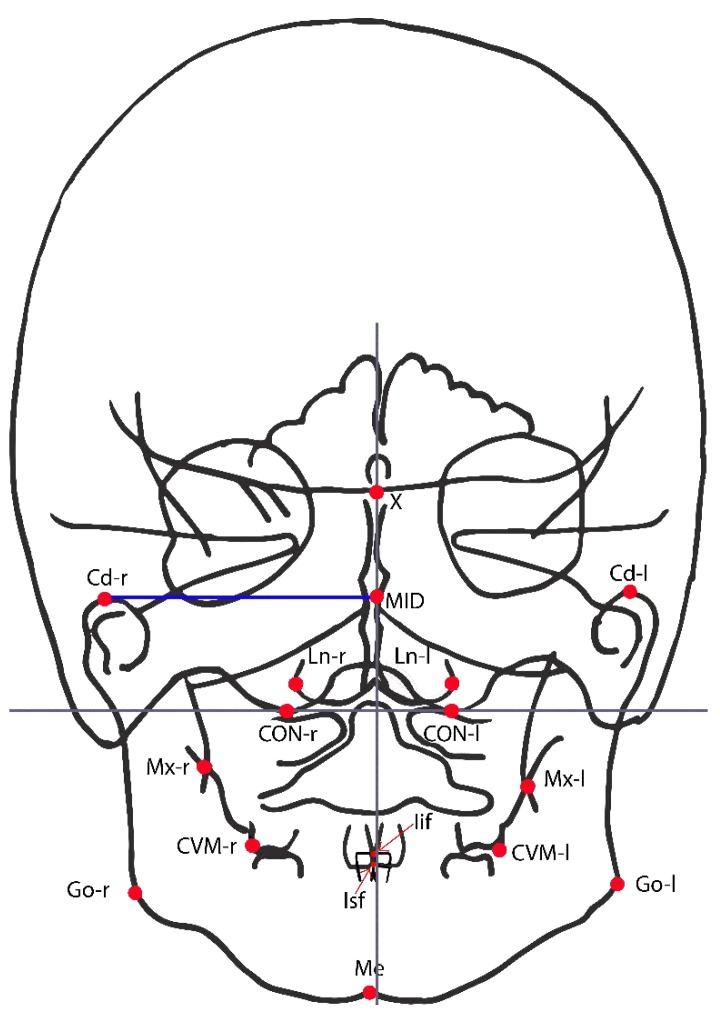
Cephalometric landmarks used in the present study on postero-anterior cephalogram. CVM+ r/l = Upper intermolar point (most prominent point on the sagittal plane of the vestibular-mesial cuspid-upper left and upper right first permanent molar); Ln r/l = Lateronasal (external points at the maximal horizontal diameter of nasal cavity); X = Crossing point between the perpendicular plate of the ethmoid and the projection of the floor of the anterior cranial fossa floor; Ans = Anterior cephalometric nasal spine; Mx r/l = Maxillare (the intersection of the lateral contour of the maxillary alveolar process and the lower contour of the maxilla-zygomatic process of the maxilla); Cd r/l = Condylar (the most superior point of the condylar head); Go r/l = Gonion (the point located at the gonial angle of the mandible); Me = Menton (is the most inferior midpoint of the chin on the outline of the mandibular symphysis); Iif = Incision inferior frontale the midpoint between the mandibular central incisors at the level of the incisal edges; Isf = Incision superior frontale (incision superior frontale, the midpoint between the maxillary central incisors at the level of the incisal edges); MID = Crossing point between the axis of symmetry and the horizontal straight line that connects the homolog cephalometric points; CON = Occipital condilion (point of cephalometric congruence between the inferior condyle of the occipital bone and the contour of the great occipital foramen); Axis of reference = Axis passing through right and left CON; Axis of symmetry = Perpendicular to the reference axis, passing through the highest point of the occipital foramen.

Fifteen points from postero-anterior cephalograms (Figure 1) were considered in the study in order to evaluate the before and after expansion in the symmetry indexes in Table 1 [33,41,42].

**Figure 2:**
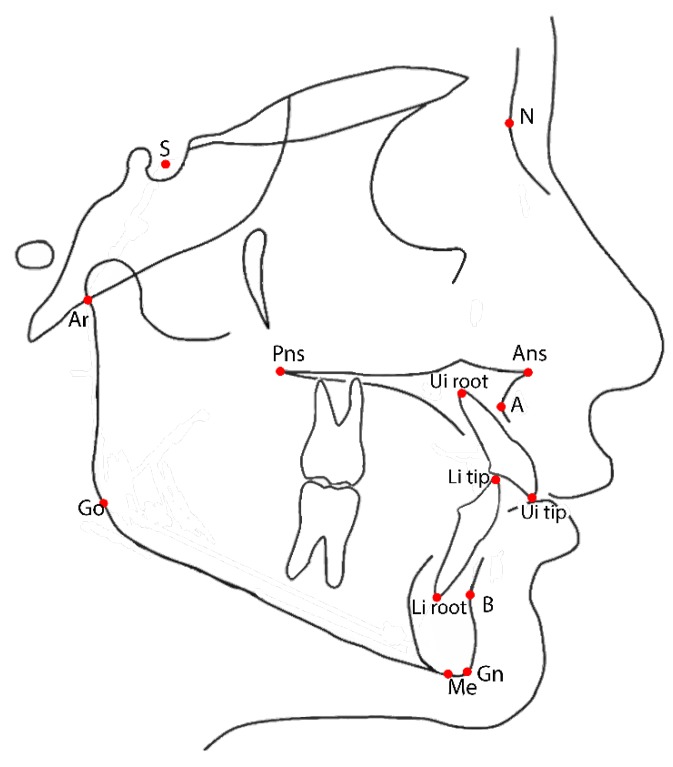
Cephalometric landmarks used in the present study on lateral cephalogram. S = Sella; N = Nasion; Ans = Anterior nasal spine; Pns = Posterior nasal spine; A = Point A (most concave point of anterior maxilla); B = Point B (most concave point of mandibular symphysis); Ui tip = Upper incisor tip; Li =lower incisor tip; Ui root = Upper incisor root; Li root = Inferior incisor root; Gn = Gnathion (midpoint between Pg and Me); Me = Menton (most inferior point of mandibular symphysis); Go = Gonion (the point located at the gonial angle of the mandible); Ar = (junction between inferior surface of the cranial base and the posterior border of the ascending rami of the mandible).

Fourteen points from lateral cephalograms (Figure 2) were considered in the study to evaluate mandibular and dental changes before and after maxillary expansion. All analyzed parameters are described in Table 1.

### 2.4. Statistical Analysis

Sample size was calculated a priori using G*Power (version 3.1.9) based on a primary outcome (SNB angle) to obtain a statistical power of the study greater than 0.80 at an alpha of 0.05, using the mean values and standard deviations of SNB with a lateral cephalometric study by Baratieri et al. [31] The values of the mean difference in SNB angle before and after maxillary expansion were used to carry out power analysis calculations along with the corresponding standard deviations. The data used to perform the analysis were: mean difference SNB = 0.78; σ = 1.26; α = 0.05; δ = 80 β = 0.2. Based on these parameters, in order to have an 80% chance of detecting a significant (at the two-sided 5% level) SNB difference between the two points in time, the sample size required was 22 patients. SPSS^®^ 23 for Windows (IBM, Sommers, NY, USA) was used for the statistical analysis.

The Shapiro–Wilk test showed that data were distributed normally. Measurements have been reported as mean and standard deviation. A paired *t*-test was applied to assess the difference before and after maxillary expansion on lateral and postero-anterior cephalograms in JIA patients and in the non-JIA control group.

An independent samples 2-tailed *t*-test was used to evaluate whether the difference before and after treatment of each parameter between the two groups was significantly different, and whether the difference between the Non-affected and Affected side in JIA group and between the right and left side in the control group were significantly different. A *p* value <0.05 was considered as statistically significant.

## 3. Results

### 3.1. Comparisons within Juvenile Idiopathic Arthritis (JIA) Patients

Descriptive statistics and statistical comparisons of postero-anterior and lateral measurements before (T0) and after treatment (T1) with RME in patients affected by JIA are shown in Table 2.

Statistically significant differences were found (*p* < 0.05) when comparing measurements on postero-anterior cephalograms both before and after RME for the following parameters: maxillary transverse dimension (Mx r-l); upper intermolar width (Cvm+ r-l); nasal cavity width (NL r-l); linear distance between the affected condyle and the axis of symmetry (Cd A-MID); linear difference between affected/non-affected condyle and the axis of symmetry (ΔCd NA/A-MID); linear distance between the non-affected gonion and the axis of symmetry (Go NA-MID); linear distance between the affected gonion and the axis of symmetry (Go A-MID); linear difference between affected/non-affected gonion and the axis of symmetry (ΔGo NA/A -MID); linear distance between menton and the axis of symmetry (Me-MID); linear distance between incision inferior frontale and the axis of symmetry (Iif-MID); linear distance between incision superior frontale and the axis of symmetry (Isf-MID) (Table 2). No statistically significant differences (*p* >0.05) were noticed for the linear distance between the non-affected condyle and the axis of symmetry (Cd NA-MID), and the linear distance between incision superior frontale and the axis of symmetry (Isf-MID) (Table 2).

Measurements on lateral cephalogram showed no statistically significant difference for all the parameters evaluated apart from ANB angle, which demonstrated a statistically significant reduction (*p* < 0.05) (Table 2).

### 3.2. Comparisons within Control Patients

Descriptive statistics and statistical comparisons of postero-anterior and lateral measurements before (T0) and after treatment (T1) with RME in the non-JIA control group are shown in Table 3.

A statistically significant difference (*p* < 0.05) has been noticed comparing postero-anterior cephalograms before and after RME in the control group for the following parameters: maxillary transverse dimension (Mx r-l); upper intermolar width (Cvm+ r-l); nasal cavity width (NL r-l); linear distance between the condyle closer to the axis of symmetry and the axis of symmetry (Cd A-MID); linear distance between incision inferior frontale and the axis of symmetry (Iif-MID). No statistically significant difference (*p* > 0.05) was found for all the other measurements on postero-anterio cephalograms (Table 3).

No statistically significant difference (*p* >0.05) has been found for all the measurements on the lateral cephalogram in the control group (Table 3).

### 3.3. Comparisons between JIA Patients and Control Group

Descriptive statistics and statistical comparisons of postero-anterior and lateral cephalometric changes induced by RME between JIA patients and the control group are shown in Table 4.

A statistically significant difference (*p* < 0.05) has been noticed comparing the effect of maxillary expansion between the two groups for: linear difference between affected and non-affected condyle and the axis of symmetry of JIA group and linear difference between the condyle more distant from and the one closer to the axis of symmetry in control patients (ΔCd NA/A-MID), linear distance between the affected gonion and the axis of symmetry in the JIA group and the homologous parameter in control group (Go A-MID), linear difference between the affected/non-affected gonion and the axis of symmetry in JIA group and the homologous parameter in control group (ΔGo NA/A-MID); linear distance between menton to the axis of symmetry (Me-MID) (Table 4). RME showed no statistically significant difference (*p* > 0.05) between the two groups for all the other measurements evaluated on postero-anterior cephalogram (Table 4).

No statistically significant difference (*p* > 0.05) has been found for all the measurements traced on lateral cephalogram comparing the effect of RME in the JIA and control groups (Table 4).

### 3.4. Comparisons between Non-Affected and Affected Side in JIA Patients and between Right and Left Side in Control Group

Descriptive statistics and statistical comparisons between the non-affected and affected sides in JIA patients and between the right and left side in the control group are shown in Table 5.

The comparison between Non affected and affected side showed a statically significant difference for Cd-MID and Go-MID at T0 and showed no statically significant difference for Cd-MID and Go-MID at T1. On the other hand, no statistically significant difference has been noticed comparing the right and left side in the control group for Cd-MID and Go-MID at T0 and T1.

### 3.5. Other Clinical Evaluations

None of the patients reported experiencing any spontaneous pain at the level of either joint during lateral excursion, protrusive excursion, unassisted maximum opening or function during all follow-up visits (immediately after expansion, 1 month, 3 months and six months after). All patients responded negatively according to RDC-TMD IIIa criteria [37]. No reoccurrence at any other joint was reported during the observation period.

## 4. Discussion

Current available evidence on interceptive orthodontic treatment in patients suffering from JIA is low [15,17,41]. Many of the published articles are case reports, case series and expert opinions [15]. Few studies evaluate larger samples [15,17]. Therefore, there is still too little evidence on orthodontic treatment in JIA patients to define evidence-based guidelines [6,14].

Resnick et al. reported the results of an international consensus conference that took place in 2019 stating that in developing patients with no or minimal active TMJ disease, interceptive functional orthodontic treatment is recommended. This therapeutic choice may help in avoiding orthognathic surgical therapy at the end of growth or at least in reducing the complexity of surgical intervention [44].

Maxillary transverse hypoplasia may be observed in patients affected by this rheumatic condition [20]. The authors decided, therefore, to evaluate the effects of RME on postero-anterior cephalograms to assess changes in mandibular asymmetry, and on latero-lateral cephalograms to assess changes in mandibular position and/or growth. Rapid maxillary expansion (RME) is considered the treatment of choice in cases of transverse maxillary hypoplasia during primary or mixed dentition [45]. It prevents the need for a surgically assisted rapid palatal expander (SARPE) or of other more invasive treatment strategies at the end of growth [46].

In the present study, a statistically significant augmentation in maxillary transverse dimensions (maxillary width, upper intermolar width and width of the nasal cavity) after RME was found both in JIA and in control subjects. The aforementioned values are comparable to those reported by previous studies that described the effect of rapid maxillary expansion appliances [47]. No relevant difference was noted between cases and controls on values regarding maxillary expansion in accordance with the authors’ expectations. Maxillary and upper airway expansion following RME treatment have several positive effects on general health which have already been described in the literature, such as improvement in nasal breathing and nasal resistances reduction [48] that can be alleged in these patients, too [49]. The response of the maxilla to RME treatment is not affected by their rheumatic condition but the additional effects of maxillary expansion on the mandible and in particular on the condyles could be particularly beneficial in these patients.

Solving cross bite and premature contact connected to dental crowding promotes asymmetry reduction and avoidance of dental interferences during function that could cause a worsening of joint inflammation and therefore of mandibular asymmetry [24,25,26,28,29,30,35,50,51,52]. Abnormal contacts between the area of upper and lower deciduous canines (i.e., cross-bite or premature contacts) for example, are the main causes of functional mandibular shift [53]. Mandibular shift caused by these conditions may, moreover, increase the risk of developing TMJ disturbances as these malocclusions often persists in permanent dentition and then through a relevant period of a patient’s life [45].

Our study on postero-anterior cephalograms evaluated whether JIA patients presented any sign of mandibular asymmetry improvement after treatment with RME. The results showed how both condyle (Cd A-MID) and gonion (Go A-MID) of the side involved in the rheumatic degeneration underwent a statistically significant improvement in their distance from the axis of symmetry. Both Δ Cd NA/A-MID and Δ Go NA/A-MID, which measure the asymmetry as the difference in linear distances from the axis of symmetry between affected and non-affected condylar and gonion points respectively, showed a statistically significant improvement (reduction) after treatment with RME. (Table 2). Taking a closer look into the data Δ Go NA/A-MID appeared improved by a greater amount if compared with Δ Cd NA/A-MID. This event is probably due to the anatomy of the TMJ i.e., two hinge joints that are connected to each other and respond to the removal of the premature contacts with a complex three-dimensional movement of rotation and translation. The linear movement of the condylar point appears reduced when compared to the Gonion points as the latter are more distant from the rotation fulcrum (i.e., TMJ) and therefore could be subjected to a greater movement [35].

The menton (Me) and the incision inferior frontale (IIF) points improved by a statistically significant amount (Table 2) as they reduced their distance to the axis of symmetry. These improvements indicate a repositioning of the mandible following RME. The positive effects are allegedly partly due to mandible repositioning after premature contact removal and partly due to skeletal growth at the level of the condyle that will hopefully continue during the residual growth period following the restoration of a correct occlusion. Understanding condylar growth response to functional stimulation promoted by mandibular repositioning [35] is worthy of future studies hopefully based on three-dimensional imaging technologies. The growth response of articular cartilages in JIA patients could indeed be impaired because of their rheumatic condition and results may differ between subjects [54,55]. Teleradiograph in lateral projection do not allow us to separately evaluate the condylar affected and non-affected side. This limitation is due to the flattening of the skull’s three-dimensional structures on a bi-dimensional film. Additionally, postero-anterior teleradiographs, albeit useful for asymmetry evaluation, do not provide information on sagittal growth of the mandible. Three-dimensional investigations, like low dose cone beam computed tomography or high definition magnetic resonance imaging [10,56,57,58], could be able in the near future to quantify precisely the amount of mandibular repositioning and mandibular growth occurring over time with little or even no radiation exposure.

Non-JIA patients did not show any statistically significant improvement for all the asymmetry indexes assessed on postero-anterior cephalograms, because they did not present any significant asymmetry prior to the expansion (Table 3). The only parameter that showed a statistically significant improvement was the distance of the incision inferior frontale (Iif) to the axis of symmetry testifying to the reduction of the mainly positional asymmetry due to the removal of the premature contacts and/or the unilateral or bilateral cross-bite after RME treatment. (Table 3) The comparison between case and control groups showed a statistically significant difference for the following parameters: Δ Cd NA/A-MID, Go A-MID, Δ Go NA/A-MID, Me-MID, which were all improved by a significantly larger amount in the JIA group (Table 4). These results are in agreement with previously reported data: asymmetry values in JIA patients shows a greater improvement as their baseline values were significantly worse compared to those in the control groups. The distance of the affected condyle to the axis of symmetry (Cd A-MID), compared with the omologous parameter in non-JIA patients, showed no significant difference. This event is apparently due to similar condylar repositioning and growth following maxillary expansion [35].

RME could also have positive effect on TMJ health of JIA patients, as the increase in condylar space and the promotion of forward repositioning of mandibular posture could reduce condylar functional stress [30] and promote mandibular growth [31].Several scientific papers have found that anterior repositioning of the mandible is likely to occur in skeletal class II patients after RME treatment [22,32]. Palatal expansion also releases the mandible to move forward, thus promoting the mandible to grow [59], helping in Class II correction, which is the most common maxillofacial characteristics of JIA patients [11,12,54,60]. Lateral teleradiographs show a statistically significant increase in the ANB angle which diminishes by an average of 1.31 degrees. In addition, SNB angle improves, albeit not in a statistically significant way, by 1 degree on average (Table 2), thus demonstrating a variable presence of a combination of dental and skeletal effects on the mandible. As already reported in non JIA patients, Hyrax expander is capable of providing a dento-skeletal expansion without modifying the intermaxillary angle (AnsPns-GoGn) which is already increased in JIA patients. Even in our study, this parameter appears not to be influenced in both groups (Table 2). Non-JIA patients demonstrated an improvement in the ANB angle, too (Table 3).

The comparison between the affected and unaffected side in the JIA group (Table 5) showed a significant difference in both time T0 and time T1. These data make up the concept described above in which the asymmetry continues to be present even at time T1 since the mere use of an expander of the palate is not able to eliminate the discrepancy between the affected and non-affected side. Despite this, there has been an important improvement between T0 and T1 in the difference between affected and unaffected side thanks to the use of RME, which has reduced the difference between the two sides from about 2.5 mm to 1.5 mm in JIA patients. The control group showed no significant differences between left and right sides at time T0 or T1 as expected by the authors in non-JIA non-asymmetric patients (Table 5).

Certain limitations of the study should be noted, including: the analysis focused only on the bidimensional radiographs, not considering the growth of the mandible because of the obvious limitations of two-dimensional cephalometric analysis.

The high significance achieved for all measurements allows us to clearly conclude that JIA patients undergoing RME have experienced benefits both in treating maxillary hypoplasia and in partially solving positional asymmetry caused by premature contacts and cross-bite. As already proven, besides promoting reduction of asymmetry indexes maxillary expansion improved mandibular growth thus reducing skeletal second class.

The number of patients included in this study, although sufficient to identify statistically significant differences, is small in order to generalize its findings. Future studies should investigate a larger sample of subjects affected by JIA considering also the subtype of JIA present.

## 5. Conclusions

Orthodontists in cooperation with pediatric rheumatologists should be trained to recognize dentofacial alterations in patients suffering from JIA. Regular clinical examination and early treatment, together with progressive monitoring of the craniofacial development, is advised. Treatment of JIA patients in the quiescent phase with maxillary constriction using RME is a suitable option for all patients. that show no signs of disease relapse at TMJ level for about 1 year. Clinical indications are the same as for non-JIA patients. In both, it was possible to observe an improvement in nasal and maxillary width. Maxillary hypoplasia and cross-bite were solved, and as additional positive effects both mandibular asymmetry and skeletal class II improved.

The most important effect of RME was the improvement of mandibular asymmetry and the resolution of mandibular shift. Further studies on RME treatment of patients affected by JIA should be carried out to thoroughly understand its three-dimensional effects on maxillofacial conditions that affects these patients as the present results appear promising.

## Figures and Tables

**Table 1:** Definition of the measurements performed in the present study [43].

Postero-Anterio Cephalogram		Lateral Cephalogram	
Mx r/l	Maxillary transverse dimension	SNA	Angle between lines S-N and N-A
CVM + r/l	First permanent molar transvers dimension.	SNB	Angle between lines S-N and N-B
Nl r/l	Maximal horizontal diameter of the nasal cavity	ANB	Angle between lines A-N and N-B
CdNA-MID	Distance of the non-affected condyle to the axis of symmetry (in control patients it is by convention the distance from the more distant one to the axis of symmetry and the axis of symmetry)	SN-GoGn	Angle between lines S-N and Go-Gn
Cd A-MID	Distance of the affected condyle to the axis of symmetry (in control patients it is by convention the distance from the closer one to the axis of symmetry and the axis of symmetry)	AnsPns-GoGn	Angle between lines Ans-Pns and Go-Gn
ΔCd NA/A-MID	Linear difference between the distance of the non-affected and affected condyle to the axis of symmetry	SN-AnsPns	Angle between lines S-N and Ans-Pns
Go NA-MID	Distance of the non-affected gonion to the axis of symmetry (in control patients it is by convention the distance from the more distant one to the axis of symmetry and the axis of symmetry)	NSAr	Angle between N-S and S-Ar (“Articular angle”)
Go A-MID	Distance of the non-affected gonion to the axis of symmetry (in control patients it is by convention the distance from the closer one to the axis of symmetry and the axis of symmetry)	SArGo	Angle between S-Ar and Ar-Go (“Articular angle”)
ΔGo NA/A-MID	Linear difference between the distance of the non-affected and affected gonion to the axis of symmetry	ArGoGn	Angle between lines Ar-Go and Go-Me (“Total gonial angle”)
Me-MID	Distance of the point menton to the axis of symmetry	NGoGn	Angle between lines N-Go and Go-Gn (“Inferior gonial angle”)
Iif-MID	Distance of the point incision inferior frontale to the axis of symmetry	ArGoN	Angle between lines Ar-Go and Go-N (“Superior gonial angle”)
Isf-MID	Distance of the point incision superior frontale to the axis of symmetry	Jarabak’ssum	Sum of total, superior and inferior gonial angle
		U1-SN	Angle between line through long axis of upper central incisor and S-N line
		U1-AnsPns	Angle between line through long axis of upper central incisor and Ans-Pns line
		IMPA	Angle between line through long axis of upper central incisor and Go-Gn line
		U1-L1	Angle between lines through long axis of upper and lower central incisors

**Table 2:** Juvenile idiopathic arthritis (JIA) patients. Mean, standard deviation (SD) and comparisons of pre-treatment (T0) and post-treatment (T1) values with paired t-test in patients affected by JIA.

Measurements	T0 (*n* = 25)		T1 (*n* = 25)		ΔT1−T0	*p* Value
	Mean	SD	Mean	SD		
**Postero-anterior cephalogram**						
Mx r/l	59.24	3.03	62.31	2.55	3.07	<0.01 *
Cvm+r/l	56.31	2.73	62.40	2.88	6.08	<0.01 *
NLr-l	26.79	3.41	29.71	3.22	2.92	<0.01 *
Cd NA-MID	45.21	2.25	46.22	2.71	1.12	NS
Cd A-MID	42.81	2.17	44.71	2.83	1.81	<0.01 *
ΔCd NA/A-MID	2.41	1.79	1.72	1.37	−0.69	0.039 *
Go NA-MID	41.92	3.26	43.15	2.56	1.23	0.043 *
Go A-MID	39.27	3.38	41.68	2.79	2.41	<0.001 *
ΔGo NA/A-MID	2.65	1.34	1.47	1.18	−1.18	0.015 *
Me-MID	2.97	1.79	1.93	1.80	−1.04	0.021 *
Iif-MID	2.23	0.85	1.13	0.71	−1.1	<0.001 *
Isf-MID	1.23	0.81	0.95	0.6	−0.28	NS
**Lateral cephalogram**						
SNA	80.08	3.74	79.7	3.25	−0.38	NS
SNB	75.14	3.72	76.12	3.35	0.98	NS
ANB	4.93	1.79	3.62	1.95	−1.36	0.035 *
SN-GoGn	35.27	4.06	35.93	4.31	0.66	NS
AnsPns-GoGn	26.83	4.77	27.18	5.04	0.35	NS
SN-AnsPns	10.45	3.48	10.55	2.79	0.27	NS
NSAr	123.99	7.78	125.04	7.14	1.05	NS
SArGo	146.14	7.15	145.5	6.29	−0.62	NS
ArGoGn	130.45	4.18	130.2	3.98	−0.45	NS
NGoGn	76.85	5.93	77.26	3.39	0.42	NS
ArGoN	53.62	3.89	52.84	3.80	−0.85	NS
Jarabak’s sum	400.08	4.37	400.45	4.69	0.43	NS
U1-SN	103.45	5.83	104.5	10.03	0.95	NS
U1-AnsPns	115.33	5.95	117.24	9.29	1.23	NS
IMPA	87.03	5.97	87.7	5.09	0.76	NS
U1-L1	129.7	9.25	128.1	10.96	1.53	NS

Abbreviations: r = right; l = left; NA = non affected; A = affected; * *p* value < 0.05; NS = not significant.

**Table 3:** Control group. Mean, standard deviation (SD) and comparisons between pre-treatment (T0) and post-treatment (T1) values with paired t-test in non-JIA control group.

Measurements	T0 (*n* = 25)		T1 (*n* = 25)		ΔT1−T0	*p* Value
	Mean	SD	Mean	SD		
**Postero-anterior cephalogram**						
Mx r/l	58.53	2.92	61.47	2.07	2.94	<0.01 *
Cvm+r/l	56.31	0.68	61.98	0.78	5.67	<0.01 *
NLr-l	27.74	2.91	31.03	2.88	3.29	<0.01 *
Cd NA-MID	46.75	1.88	48.33	2.19	1.58	NS
Cd A-MID	47.56	2.07	49.02	2.36	1.46	NS
ΔCd NA/A-MID	0.81	1.09	0.69	0.97	−0.12	NS
Go r-MID	44.22	1.95	45.55	1.84	1.28	NS
Go l-MID	43.43	2.68	44.95	1.93	1.52	NS
ΔGo r/l-MID	0.77	0.84	0.55	1.18	−0.22	NS
Me-MID	0.72	1.37	0.33	1.51	−0.39	NS
Iif-MID	1.05	1.85	0.26	1.74	−0.75	0.042 *
Isf-MID	1.03	0.81	0.73	0.6	−0.32	NS
**Lateral cephalogram**						
SNA	83.52	4.99	83.26	5.12	−0.26	NS
SNB	78.48	6.47	79.03	6.15	0.55	NS
ANB	6.24	2.95	5.22	2.37	−1.02	0.047 *
SN-GoGn	34.96	6.07	34.61	5.98	−0.34	NS
AnsPns- GoGn	27.74	4.74	27.83	5.87	0.09	NS
SN- AnsPns	7.61	2.84	7.04	2.99	−0.56	NS
NSAr	124.00	5.66	124.13	5.36	0.13	NS
SArGo	139.91	6.37	141.09	6.15	1.17	NS
ArGoGn	132.61	5.95	131.43	4.81	−1.18	NS
NGoGn	76.91	4.91	76.04	4.43	−0.87	NS
ArGoN	55.78	4.01	55.39	4.49	−1.92	NS
Jarabak’s sum	396.52	6.78	396.61	5.84	0.09	NS
U1-SN	103.61	9.07	105.74	8.34	2.13	NS
U1-AnsPns	111.00	10.42	112.39	8.73	1.39	NS
IMPA	89.57	6.07	90.26	7.81	0.70	NS
U1-L1	129.39	9.35	127.78	8.64	−1.61	NS

Abbreviations: r = right; l = left; NA = more distant side to the axis of symmetry; A = closer side to the axis of symmetry; * *p* value < 0.05; NS = not significant.

**Table 4:** Jia vs Control group. Mean, standard deviation (SD) of measurements between T1−T0 and comparisons of values within JIA group and control group with independent *t*-test.

Measurements	JIA ΔT1−T0 (*n* = 25)		ControlΔT1−T0 (*n* = 25)		ΔJIA-Control (mean)	*p* Value
	Mean	SD	Mean	SD		
**Postero-anterior cephalogram**						
Mx r/l	3.07	2.19	2.94	1.84	0.13	NS
Cvm+r/l	6.08	3.77	5.67	2.86	0.41	NS
NLr-l	2.92	2.32	3.29	2.21	0.37	NS
Cd NA-MID	1.12	2.33	1.58 ^A^	1.87	-0.46	NS
Cd A-MID	1.81	2.15	1.46 ^B^	1.98	0.35	NS
ΔCd NA/A-MID	−0.69	1.72	−0.12 ^A,B^	0.66	-0.57	0.034 *
Go NA-MID	1.23	2.12	1.28 ^A^	1.25	-0.05	NS
Go A-MID	2.41	1.52	1.53 ^B^	1.16	0.89	0.026 *
ΔGo NA/A-MID	−1.18	1.49	−0.22 ^A,B^	1.67	-0.96	0.037 *
Me-MID	−1.04	0.93	−0.39	0.85	0.65	0.013 *
Iif-MID	−1.1	0.66	−0.75	0.43	-0.35	NS
Isf-MID	−0.28	0.59	−0.32	0.69	0.04	NS
**Lateral cephalogram**						
SNA	−0.38	3.73	−0.26	5.03	0.12	NS
SNB	0.98	2.95	0.35	6.98	−0.63	NS
ANB	−1.36	1.73	−1.02	2.50	0.34	NS
SN-GoGn	0.66	1.77	−0.34	2.67	−1	NS
AnsPns-GoGn	0.35	4.48	0.09	3.22	−0.36	NS
SN-AnsPns	0.27	4.82	−0.56	2.95	−0.83	NS
NSAr	1.05	3.03	0.13	4.98	−0.92	NS
SArGo	−0.62	8.52	1.17	5.52	1.79	NS
ArGoGn	−0.45	4.71	−1.18	3.99	−0.73	NS
NGoGn	0.42	3.84	−0.87	2.60	1.29	NS
ArGoN	−0.85	3.62	−1.92	3.54	−1.07	NS
Jarabak’s sum	0.43	1.68	0.09	3.42	−0.34	NS
U1-SN	0.95	7.43	2.13	9.79	1.18	NS
U1-AnsPns	1.23	7.26	1.39	10.31	0.16	NS
IMPA	0.76	5.24	0.70	5.73	−0.06	NS
U1-L1	1.53	6.51	−1.61	8.91	−3.14	NS

Abbreviations: r = right; l = left; NA = non affected side (JIA group)/more distant side to the axis of symmetry (control group); A = affected side (JIA group)/ closer side to the axis of symmetry (control group); ^A^ = in control patients it is by convention the distance from the more distant one to the axis of symmetry and the axis of symmetry; ^B^ = in control patients it is by convention the distance from the closer one to the axis of symmetry and the axis of symmetry; * *p* value < 0.05; NS = not significant.

**Table 5:** Mean, standard deviation (S.D.) and comparisons of pre-treatment (T0) and post-treatment (T1) values with independent *t*-test between Non-affected and Affected side in JIA group and between right and left side in non-JIA control group.

**Measurements**	**Non-Affected**		**Affected**		**Δ Non Affected–Affected**	***p* Value**
	**Mean**	**SD**	**Mean**	**SD**		
**JIA patients**						
Cd-MID T0	45.21	2.25	42.81	2.17	2.41	<0.001 *
Cd-MID T1	46.22	2.71	44.71	2.83	1.51	NS
Go-MID T0	41.92	3.26	39.27	3.38	2.65	0.007 *
Go-MID T1	43.15	2.56	41.68	2.79	1.46	NS
**Measurements**	**Right**		**Left**		**Δ Non affected- Affected**	***p*** **value**
	**Mean**	**SD**	**Mean**	**SD**		
**Control group**						
Cd-MID T0	46.75	1.88	47.56	2.07	−0.81	NS
Cd-MID T1	48.33	2.19	49.02	2.36	−0.69	NS
Go-MID T0	44.22	1.95	43.43	2.68	0.79	NS
Go-MID T1	45.55	1.84	44.95	1.93	0.61	NS

Abbreviations: r = right; l = left; * *p* value < 0.05; NS = not significant.
